# Effectiveness of AI-Based Tools in Detecting Diabetic Retinopathy in Low- and Middle-Income Countries: A Systematic Review of Diagnostic Performance and Implementation Feasibility

**DOI:** 10.7759/cureus.96554

**Published:** 2025-11-11

**Authors:** Nneoma Onyeze, Sami Sartawi, Zain Nayyer

**Affiliations:** 1 Surgery, Ninewells Hospital, Dundee, GBR; 2 Oral and Maxillofacial Surgery, Ninewells Hospital, Dundee, GBR; 3 Emergency Medicine, Ninewells Hospital, Dundee, GBR

**Keywords:** artificial intelligence, cost-effectiveness, diabetic retinopathy, health systems, implementation feasibility, low- and middle-income countries (lmics), screening

## Abstract

Diabetic retinopathy (DR) is a leading cause of preventable blindness worldwide, with a disproportionate impact in low- and middle-income countries (LMICs). Artificial intelligence (AI) offers a potential means to address workforce and infrastructure gaps that limit access to DR screening in these settings, but evidence on its performance and feasibility remains scattered. A systematic review of studies published between January 2015 and June 2025 was conducted using six databases. Eligible studies evaluated AI, machine learning, or deep learning applied to retinal imaging for DR detection and reported quantitative diagnostic or implementation outcomes, while studies limited to high-income countries or non-original research were excluded. Only a small number of eligible studies were identified. Across these, AI-based tools generally showed high diagnostic accuracy and were feasible to implement in resource-limited environments. Early evidence suggested potential benefits, such as reduced screening costs, decreased clinician workload, and improved patient follow-up, though reporting on infrastructure needs, regulatory considerations, and long-term sustainability was limited. Overall, AI-based tools show promise for scaling DR screening in LMICs, with encouraging indications of good accuracy and operational efficiency, but further large-scale and implementation-focused research is required to guide their integration into health systems.

## Introduction and background

Diabetic retinopathy (DR) is a leading cause of preventable blindness worldwide and one of the most serious complications of diabetes mellitus. The global prevalence of diabetes has risen steadily, with the sharpest increases occurring in low- and middle-income countries (LMICs), where more than three-quarters of people with diabetes now reside [1]. Throughout, we use the World Bank analytical income groups - low, lower-middle, and upper-middle income-based on gross national income (GNI) per capita (Atlas method). Classifications are fixed for the fiscal year; we anchor to FY25 (July 1, 2024-June 30, 2025). DR affects roughly one in three individuals with diabetes, and without timely detection and treatment, it can lead to irreversible vision loss [2]. Screening and early intervention are therefore essential to reducing blindness from DR.

Despite this urgent need, implementing systematic screening programs in LMICs remains challenging. Many countries face a shortage of trained ophthalmologists, limited access to advanced retinal imaging equipment, and insufficient infrastructure to support large-scale eye care services [3,4]. Traditional screening strategies that depend on specialist interpretation of fundus images are resource-intensive and often impractical in these contexts. As a result, large numbers of individuals with diabetes in LMICs remain unscreened, leading to late presentation and avoidable visual impairment [5].

Artificial intelligence (AI) has emerged as a promising solution to these barriers. Advances in machine learning and deep learning have enabled the development of algorithms capable of detecting DR from retinal images with diagnostic accuracy comparable to expert ophthalmologists [6-8]. Landmark studies have demonstrated strong performance, with sensitivities and specificities exceeding 85% for referable DR [6,7], and the first autonomous AI diagnostic system was successfully trialled in primary care in the United States [8]. Meta-analyses further confirm pooled sensitivity and specificity values above 85% across diverse datasets [9,10].

Evidence from LMICs is now beginning to accumulate. A recent scoping review identified a growing number of AI-based DR screening studies in these settings, though many remain at the pilot stage [1]. In India, the Artificial Intelligence Diabetic Retinopathy Screening System (AIDRSS) achieved 92% sensitivity and 88% specificity on more than 10,000 images, with 100% sensitivity for vision-threatening DR [2]. Validation studies in African populations have also demonstrated reliable performance, suggesting that AI can be adapted to resource-limited environments [7]. These findings highlight the potential of AI tools to expand screening coverage in regions where access to ophthalmologists is limited.

Beyond diagnostic accuracy, implementation outcomes are especially important for LMICs. Cost-effectiveness analyses indicate that AI-based screening may reduce expenses compared with human grading, with one study reporting per-patient savings of more than USD 140 while maintaining 100% sensitivity [3]. AI-assisted screening has also been shown to improve patient adherence to follow-up appointments, an important component of care pathways [11,12]. Workflow studies suggest that AI can reduce clinician workload and streamline screening processes, though challenges remain in infrastructure, regulatory approval, and data governance [13-15].

Concerns have also been raised regarding fairness, bias, and generalizability. Many algorithms are trained on datasets from high-income countries, which may not adequately reflect disease presentation or image quality in LMICs [16,17]. Recent efforts have sought to address these challenges through responsible AI frameworks that emphasize equity, transparency, and adaptability [18].

Although several reviews have summarized the global application of AI for DR screening [16], none have comprehensively evaluated both diagnostic performance and implementation feasibility in LMICs. Addressing this gap is essential to guide policymakers, clinicians, and researchers in developing scalable, cost-effective, and equitable AI-based screening programs. The aim of this systematic review is therefore to evaluate the effectiveness of AI-based tools in detecting DR in LMICs. Specifically, it synthesizes evidence on diagnostic accuracy, including sensitivity, specificity, and area under the curve, and assesses implementation feasibility in terms of cost, workflow integration, and acceptability. By addressing both performance and feasibility, this review provides a timely appraisal of the role of AI in reducing the burden of DR in resource-limited settings.

## Review

Method

Eligibility Criteria

We included studies from countries classified by the World Bank as low, lower-middle, or upper-middle income in FY25. Country income status was taken from the World Bank “Country and Lending Groups” page as of July 1, 2024, which applied artificial intelligence (AI), machine learning, or deep learning approaches to retinal imaging for the detection of DR, as these study designs provide the most reliable evidence of diagnostic accuracy and feasibility in real-world contexts. Both community-based and hospital-based screening settings were eligible because AI tools may be implemented at different levels of care, from primary screening programs to tertiary referral centres. To ensure the quality and accessibility of evidence, only English-language articles published in peer-reviewed journals and high-quality preprints available in reputable repositories were considered. We excluded case reports, narrative reviews, editorials, and commentaries because they do not provide primary data suitable for synthesis; we also excluded studies conducted outside LMICs since the focus of this review is on resource-limited settings where implementation challenges are most pressing. Finally, we excluded studies that did not report quantitative outcomes on diagnostic performance, such as sensitivity, specificity, or area under the curve or implementation outcomes, such as cost or feasibility, as these are essential for evaluating both effectiveness and practical applicability. The FY25 thresholds (GNI per capita, 2023 US$) are as follows: low ≤ $1,135; lower-middle $1,136-$4,495; and upper-middle $4,496-$13,935.

Information Sources and Search Strategy

A comprehensive literature search was performed across major biomedical and technical databases, including PubMed/MEDLINE, Embase, Scopus, Web of Science, IEEE Xplore, and the Cochrane Library, covering all available records from January 2015 through June 2025. These databases were selected to capture both clinical ophthalmology research and computer science studies in AI. In addition, preprint repositories such as arXiv were searched to identify high-quality emerging evidence, recognizing that AI-related work often appears first in preprints before peer-reviewed publication. The search strategy combined controlled vocabulary terms and free-text keywords related to diabetic retinopathy, artificial intelligence, machine learning, deep learning, and low- and middle-income countries. For example, the PubMed search included the following terms: (“diabetic retinopathy” AND (“artificial intelligence” OR “machine learning” OR “deep learning”)) AND (“low-income countries” OR “middle-income countries” OR “resource-limited settings”). Search strategies were adapted for each database to account for variations in indexing. To ensure completeness, the reference lists of all included studies and relevant reviews were also manually screened for additional eligible publications. Country income group for each study was verified against the World Bank's Country and Lending Groups’ list for FY25 at data extraction.

Selection Process

All search results were imported into reference management software to facilitate organization and duplicate removal. Titles and abstracts of the retrieved records were screened independently by two reviewers against the predefined eligibility criteria to ensure consistency in study selection. Full texts of potentially relevant articles were then retrieved and assessed in detail by the same reviewers to confirm eligibility. At each stage, disagreements were resolved through discussion, and when consensus could not be reached, a third reviewer was consulted to provide arbitration. This multi-step process was adopted to minimize selection bias and to ensure that only studies directly relevant to the research question were included. The overall process will be documented in a PRISMA flow diagram, which will summarize the number of studies identified, screened, included, and excluded, along with the reasons for exclusion at the full-text stage.

Data Extraction

Two reviewers independently extracted data from each included study using a standardized form. Extracted information included study details (author, year, country, design, setting), population characteristics (sample size, demographics), imaging modality, type of AI model, and whether external validation was performed. Outcomes of interest were diagnostic performance measures (sensitivity, specificity, accuracy, area under the curve) and implementation outcomes (cost-effectiveness, feasibility, integration into healthcare systems, acceptability). Study limitations and potential sources of bias were also recorded. Disagreements were resolved through discussion or consultation with a third reviewer.

Risk of Bias Assessment

The risk of bias in included studies was independently assessed by two reviewers. Diagnostic accuracy studies were evaluated using the QUADAS-2 tool, while studies reporting prediction models were assessed with PROBAST. Any disagreements were resolved through discussion or by consulting a third reviewer.

Results

Study Selection

**Figure 1:**
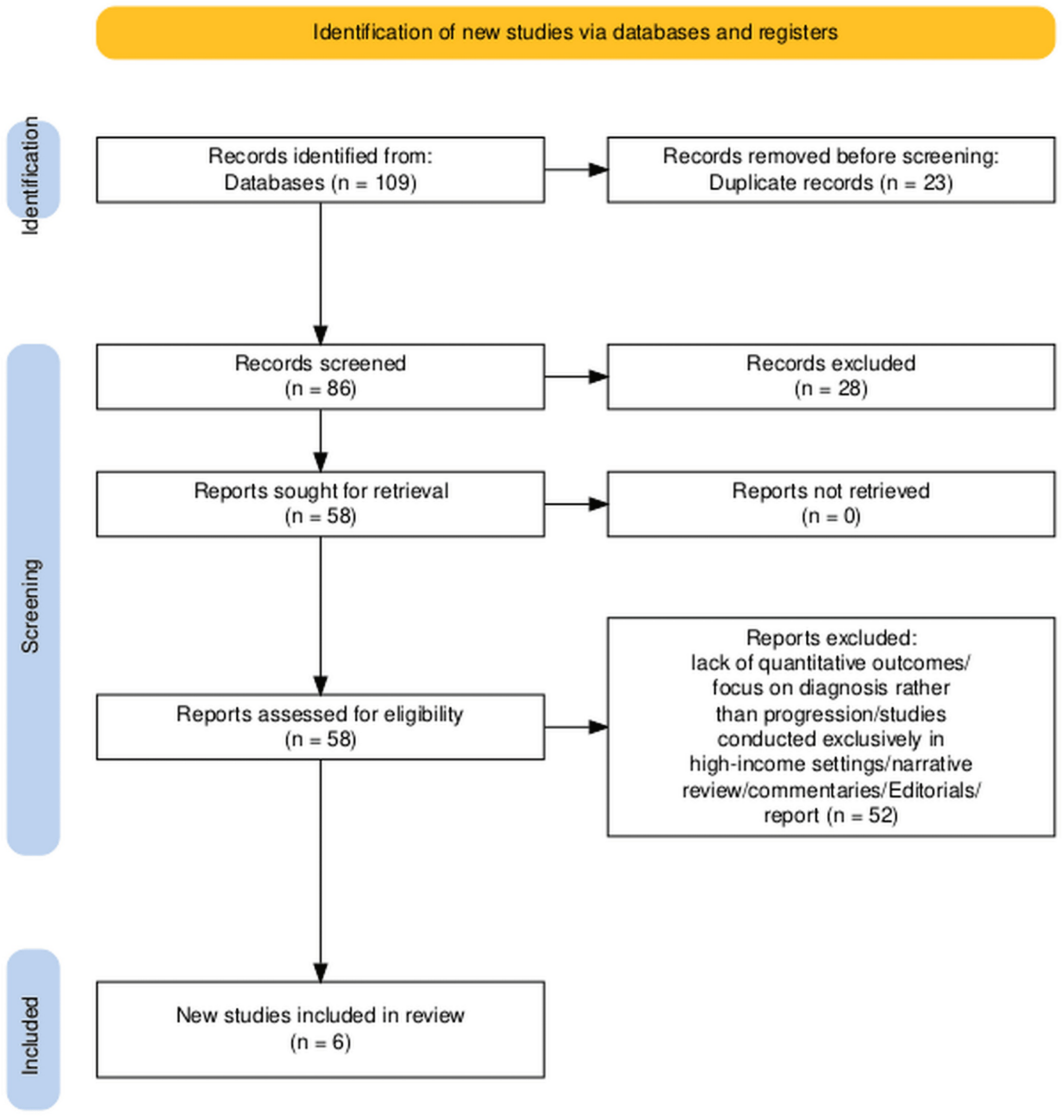
PRISMA flow chart depicting the study selection PRISMA: Preferred Reporting Items for Systematic Reviews and Meta-Analysis [21]

The initial search retrieved 109 records from all databases. After the removal of 23 duplicates, 86 unique records were screened by title and abstract. Of these, 58 full-text articles were assessed for eligibility. At the full-text stage, 52 studies were excluded for the following reasons: lack of quantitative outcomes, focus on diagnosis rather than progression, and studies conducted exclusively in high-income settings. A total of six studies met the predefined inclusion criteria and were included in this systematic review [2,3,7,15,19,20]. The selection process is summarized in the Preferred Reporting Items for Systematic Reviews and Meta-Analysis (PRISMA) flow diagram (Figure 1).

Study Characteristics

We included six studies conducted across five LMICs (FY25 World Bank groups): India (two studies), Zambia, Thailand, Rwanda, and Brazil. By income tier, settings were distributed as follows: low-income (1; Rwanda), lower-middle-income (3; India ×2, Zambia), and upper-middle-income (2; Thailand, Brazil). Core design features, settings, sample sizes, imaging modalities, and AI systems are summarized in Table 1, while diagnostic/implementation outcomes are summarized in Table 2. Two studies were prospective clinical/multicenter validations (India; Zambia), one was a national program, a prospective interventional cohort (Thailand), one was a randomized controlled trial (RCT) evaluating implementation impact (Rwanda), one a pilot feasibility study in primary care (Brazil), and one was a cross-sectional prospective validation using smartphone-based non-mydriatic imaging (India). Studies were conducted in primary-care clinics, national screening sites, and rural/low-resource outpatient settings, reflecting real-world pathways for DR screening (Table 1). Sample sizes ranged from n=275 (implementation RCT in Rwanda) to n=7,651 (Thai national program), with image volumes reported where applicable (e.g., 10,058 images in India; 4,504 images in Zambia). AI systems included the following: AIDRSS (India), a bespoke deep-learning algorithm (Zambia), the Google/Thai Ministry of Public Health DL system (Thailand), an AI triage workflow with immediate feedback (Rwanda), embedded AI on a handheld fundus camera (Phelcom Eyer) (Brazil), and offline Medios AI paired with a Remidio smartphone fundus camera (India). Comparators were rigorous and varied - ophthalmologist grading, adjudicated retina specialists, consensus panels, delayed human grading, and remote specialist over-reads - supporting external validity (Table 1). Across validations, AI achieved high diagnostic performance for referable and/or vision-threatening DR, with reported metrics including sensitivity up to ~92-100% and specificity ~88-95%; the Thai program reported accuracy ~94.7% with strong sensitivity/specificity. Implementation-oriented studies demonstrated operational feasibility (Brazil) and meaningful pathway effects, including a +11.9% absolute increase in referral uptake with AI-augmented triage in Rwanda. Detailed point estimates (sensitivity, specificity, accuracy), confidence intervals (CIs) where reported, and implementation outcomes (e.g., same-visit referral capability, real-time triage) are presented in Table 2. Anchoring to FY25 World Bank income groups, the evidence spans low-, lower-middle-, and upper-middle-income contexts, with two studies in upper-middle-income economies (Brazil, Thailand), three in lower-middle-income settings (India ×2, Zambia), and one in a low-income setting (Rwanda). This spread, alongside varied care levels and imaging hardware (handheld/smartphone vs tabletop), supports the generalizability of AI-enabled DR screening across resource levels (Tables 1-2).

**Table 1:** Characteristics of the included studies evaluating AI-based tools for diabetic retinopathy screening in low- and middle-income countries (LMICs) This table summarizes six eligible studies assessing AI-based tools for diabetic retinopathy detection in LMICs or LMIC-applicable settings, detailing author, year, country, design, sample size, AI system, comparator, and key outcomes, such as diagnostic accuracy and implementation metrics. AIDRSS: Artificial Intelligence Diabetic Retinopathy Screening System; DME = diabetic macular edema; DR = diabetic retinopathy; LMIC = low- and middle-income country

Author (Year)	Country	Design	Sample Size	AI Tool	Comparator	Key Outcomes
Dey et al. (2025) [2]	India	Prospective multicenter validation	5,029 participants; 10,058 images	AIDRSS	Ophthalmologist grading	Sensitivity 92%, specificity 88%; 100% sensitivity for vision-threatening DR
Bellemo et al. (2019) [7]	Zambia (LMIC)	Prospective clinical validation	1,574 patients; 4,504 images (3,093 eyes)	Deep learning algorithm	Ophthalmologist grading	Reliable detection of referable and vision-threatening DR
Ruamviboonsuk et al. (2022) [3]	Thailand	Prospective interventional cohort (national program)	7,651 participants (nine primary-care sites)	DL system (Google/Thai MoPH)	Adjudicated retina specialists	VTDR: accuracy 94.7%, sensitivity 91.4%, specificity 95.4%; real-time referrals
Mathenge et al. (2022) [15]	Rwanda	Randomized controlled trial (implementation impact)	n=275 randomized after AI-positive screen	AI triage with immediate feedback	Delayed human grading (3–5 days)	Referral uptake 51.5% vs 39.6% (absolute +11.9%; P=0.048)
Malerbi et al. (2022) [19]	Brazil	Pilot feasibility of AI-enabled DR screening with portable camera (primary care)	1,046 screened in rural/low-resource clinics	Embedded AI on handheld fundus camera (Phelcom Eyer)	Remote retinal specialist over-read	Feasible workflow; high gradability; enabled same-visit triage/referral
Sosale et al. (2020) [20]	India	Cross-sectional prospective validation using smartphone-based non-mydriatic imaging	900 analyzed (from 922 enrolled)	Medios offline AI (Remidio FOP camera)	Consensus of five retina specialists	Referable DR: sensitivity 93%, specificity 92.5%

**Table 2:** Country income classification (World Bank, FY25) For each study, the primary operating point (percent; 95% CI where available) for referable DR and, when reported, VTDR, grouped by country and World Bank FY25 income tier, with per-patient results preferred over per-eye. Se/Sp = sensitivity/specificity; Acc = accuracy; AUC = area under ROC; PPV/NPV = positive/negative predictive value; VTDR = vision-threatening DR

Study	Country	WB income group (FY25)	Source
Dey et al. (2025) [2]	India	Lower-middle income	World Bank “ Country & Lending Groups (FY25)
Bellemo et al. (2019) [7]	Zambia	Lower-middle income	World Bank “ Country & Lending Groups (FY25)
Ruamviboonsuk et al. (2022) [3]	Thailand	Upper-middle income	World Bank “ Country & Lending Groups (FY25)
Mathenge et al. (2022) [15]	Rwanda	Low income	World Bank “ Country & Lending Groups (FY25)
Malerbi et al. (2022) [19]	Brazil	Upper-middle income	World Bank “ Country & Lending Groups (FY25)
Sosale et al. (2020) [20]	India	Lower-middle income	World Bank “ Country & Lending Groups (FY25)

Diagnostic Accuracy

Across six LMIC studies, AI systems showed consistently high diagnostic performance for detecting referable and vision-threatening DR, but incomplete or non-uniform CIs and missing 2×2 data precluded pooling. In India, AIDRSS achieved 92% sensitivity and 88% specificity, with 100% sensitivity for vision-threatening DR (VTDR), in a large multicentre cohort (5,029 participants; 10,058 images) (CIs not reported in the source extract) [2]. In Thailand’s national screening programme, a deployed Google/Thai MoPH system maintained high performance for VTDR accuracy 94.7% (95% CI: 93.0-96.2), sensitivity 91.4% (95% CI: 87.1-95.0), specificity 95.4% (95% CI: 94.1-96.7), and supported same-visit referrals [3]. Community screening in India using a smartphone, non-mydriatic camera (Remidio FOP) with Medios (offline) reported referable DR sensitivity of 93.0% (95% CI: 91.3-94.7) and specificity of 92.5% (95% CI: 90.8-94.2) against a five-specialist consensus [20]. In Zambia, a prospective clinical validation reported AUC of 0.973 (95% CI: 0.969-0.978), with sensitivity of 92.25% (95% CI: 90.10-94.12) and specificity 89.04% (95% CI: 87.85-90.28) for referable DR, indicating reliable detection of both referable and vision-threatening DR [7]. A Brazilian pilot using a handheld camera with embedded AI reported high image gradability and same-visit triage, reinforcing real-world feasibility, though without full accuracy matrices for pooling [19]. The RAIDERS randomized trial in Rwanda primarily evaluated implementation impact (increased referral uptake with immediate AI feedback) rather than head-to-head accuracy, but it underscores the clinical utility of rapid AI-supported triage in low-resource pathways [15]. Taken together, studies reported explicit metrics cluster around high sensitivity (~91-93%) and high specificity (~88-95%) across devices and care levels; however, heterogeneity in reference standards, thresholds, and handling of ungradable images and the absence of consistent CIs or 2×2 counts limits comparability and justifies a narrative (non-meta-analytic) synthesis and call for standardized reporting (TP/FP/FN/TN and CIs) to enable future quantitative aggregation.

Implementation and Feasibility

In practice, programs that rolled out AI largely preserved the strong diagnostic accuracy seen in validation and turned it into workable care pathways. Thailand’s national screening program integrated a deployed DL system that kept high performance for VTDR while enabling real-time, same-visit referrals [3]. In Rwanda, the RAIDERS randomized trial showed a +11.9% absolute gain in referral uptake with AI-supported triage (51.5% vs 39.6%; p = 0.048) - evidence that faster feedback can translate into more patients reaching care [15]. Primary-care pilots using handheld or smartphone fundus cameras in Brazil and India reported high image gradability and same-visit triage, reducing downstream manual review and aligning with the accuracy levels reported in diagnostic studies [19,20]. However, most accounts gave limited detail on infrastructure needs, integration steps, ongoing QA, and long-term sustainability - gaps that should be addressed to support scale-up (Tables 1-2).

Ethical and Equity Considerations

Most algorithms were originally trained on data from high-income settings, raising concerns about domain shift and fairness when applied in LMIC populations with different imaging conditions and disease distributions [16,17]. Subgroup performance (by device type, image quality, or demographic factors) was seldom reported. Frameworks such as RAIS-DR offer practical guidance on transparency, bias assessment, and equity safeguards, but uptake in the included literature was limited [18].

Risk of Bias

Overall risk of bias was moderate. Common limitations included narrow external validation, variability in reference standards (single graders vs adjudication), and non-uniform definitions of DR severity. Only a few studies were prospective multicentre evaluations, and just one used a randomized design, which tempers generalizability across LMIC contexts. We interpret effect estimates with these design features in mind (see Table 1) and emphasize the study-level metrics and confidence intervals where available (see Table 2).

Discussion

Diagnostic Performance of AI for DR

Across the six included studies, AI systems delivered specialist-approaching screening performance for referable and, in several cases, vision-threatening DR across diverse workflows and devices. The two Indian validations (AIDRSS; Medios with smartphone imaging) reported high sensitivity and specificity, the Zambian study showed robust performance under routine clinic conditions, and Thailand’s national programme demonstrated high accuracy at scale with same-visit referral support [2,3,7,20]. Together, these findings support AI as a credible front-end screening/triage tool in LMICs, while gaps in 2×2 reporting and CIs argue for improved standardization to enable future quantitative synthesis.

Validation and Early Evidence From LMICs

Although the evidence base is still modest, it spans diverse LMIC contexts: lower-middle-income settings (India ×2, Zambia), an upper-middle-income national program (Thailand), an upper-middle-income primary-care rollout (Brazil), and a low-income randomized trial (Rwanda). Performance was generally robust even where infrastructure is limited, and studies that used locally captured data suggest models that can adapt well to new populations when workflows and image acquisition are standardized [2,3,7,19,20]. That said, most LMIC studies remain small, site-specific, and short in duration, which limits external validity and makes it hard to judge the longevity of performance.

Feasibility and Real-World Deployment of AI Systems

Implementation findings are encouraging. Rwanda’s randomized trial showed a meaningful improvement in referral completion with AI-supported triage (absolute +11.9%; 51.5% vs 39.6%; p = 0.048) [15]. Brazil’s primary-care study demonstrated that embedded AI on a handheld camera can yield high gradability and enable same-visit decisions in rural clinics [19]. The Thai program illustrated how AI can be integrated at a national scale to trigger real-time referrals [3]. India’s smartphone-based, largely offline workflow points to practical options where connectivity is unreliable [20]. Even so, key operational details - power backup, device maintenance, training and supervision of non-specialist staff, data flows, and integration with referral pathways - were only partially reported. These factors will determine whether early gains translate into durable service improvements (see Tables 1-2).

Ethical, Equity, and Governance Considerations

Most systems were originally developed or trained in high-income settings. Without careful local validation and ongoing monitoring, there is a risk of domain shift and uneven performance across subgroups. Few included studies reported stratified results by device, image quality, age, sex, or other equity-relevant characteristics. Responsible deployment in LMICs should therefore include transparent reporting, local fine-tuning where needed, clear escalation rules for ungradable images, and plans for post-deployment auditing to detect drift and bias.

Implications and Future Directions

For health systems with limited specialist capacity, AI can expand screening reach, speed up decision-making, and support more reliable referral pathways. The six studies sketch workable models: point-of-care triage with same-visit action (Brazil, India), program-wide integration with real-time referrals (Thailand), and randomized evidence of pathway impact (Rwanda). Pairing these approaches with teleophthalmology and standardized referral protocols could help bring earlier detection to rural and underserved communities.

Strengths and Limitations of Current Evidence

The review brings together prospective validations, a national deployment, a randomized trial, and primary-care implementations across five countries and three income tiers, offering a realistic picture of what AI-enabled DR screening can achieve in LMICs. However, the evidence remains limited by small sample sizes outside Thailand, short follow-up, heterogeneous reference standards and outcome definitions, and sparse reporting on infrastructure and integration. Equity analyses were rare. Next steps should prioritize larger, multicentre, prospective LMIC studies with standardized protocols; equity-focused subgroup reporting; and detailed implementation metrics (e.g., time-to-referral, linkage to care, workforce impact, costs) to guide sustainable scale-up.

## Conclusions

This systematic review highlights the promise of AI in diabetic retinopathy screening across LMICs, where shortages of specialists and infrastructure pose significant barriers to care. The strengths of this review include a comprehensive search across multiple databases, strict eligibility criteria, and adherence to PRISMA guidelines. By focusing only on studies that reported quantitative outcomes from LMICs, it provides evidence most relevant to real-world needs. The inclusion of both diagnostic accuracy and implementation outcomes further ensures a balanced appraisal of clinical and operational feasibility.

However, the review is limited by the small number of eligible studies, restricted geographic representation, and heterogeneity in study design and outcome reporting, which precluded formal meta-analysis. Some included studies were preprints or early-phase pilots, which may be subject to revision. Despite these limitations, the findings consistently demonstrate high diagnostic accuracy of AI-based tools, along with early evidence of cost-effectiveness, workload reduction, and improved patient adherence. To build on this progress, future research should prioritize large multicentre trials in diverse LMIC settings, standardization of reporting, and long-term implementation studies. Policymakers should simultaneously invest in infrastructure, regulatory frameworks, and integration with teleophthalmology to enable safe, equitable, and sustainable deployment of AI, ultimately reducing preventable blindness from diabetic retinopathy.

## References

[1] Cleland CR, Rwiza J, Evans JR, Gordon I, MacLeod D, Burton MJ, Bascaran C (2023). Artificial intelligence for diabetic retinopathy in low-income and middle-income countries: a scoping review. BMJ Open Diabetes Res Care.

[2] Dey AK, Walia P, Somvanshi G, Ali A, Das S, Paul P, Ghosh M (2025). AI-driven diabetic retinopathy screening: multicentric validation of AIDRSS in India [PREPRINT]. arXiv.

[3] Ruamviboonsuk P, Tiwari R, Sayres R (2022). Real-time diabetic retinopathy screening by deep learning in a multisite national screening programme: a prospective interventional cohort study. Lancet Digital Health.

[4] Gulshan V, Peng L, Coram M (2016). Development and validation of a deep learning algorithm for detection of diabetic retinopathy in retinal fundus photographs. JAMA.

[5] Ting DS, Cheung CY, Lim G (2017). Development and validation of a deep learning system for diabetic retinopathy and related eye diseases using retinal images from multiethnic populations with diabetes. JAMA.

[6] Abràmoff MD, Lavin PT, Birch M, Shah N, Folk JC (2018). Pivotal trial of an autonomous AI-based diagnostic system for detection of diabetic retinopathy in primary care offices. NPJ Digit Med.

[7] Bellemo V, Lim ZW, Lim G (2019). Artificial intelligence using deep learning to screen for referable and vision-threatening diabetic retinopathy in Africa: a clinical validation study. Lancet Digital Health.

[8] Rahmati M, Smith L, Piyasena MP (2025). Artificial Intelligence improves follow-up appointment uptake for diabetic retinal assessment: a systematic review and meta-analysis. Eye (Lond).

[9] DeLuca NJ, Wertheimer B, Ansari Z (2025). Artificial intelligence in ophthalmic screening: advancing diabetic retinopathy detection in low-income immigrant populations. Curr Ophthalmol Rep.

[10] Alqahtani AS, Alshareef WM, Aljadani HT, Hawsawi WO, Shaheen MH (2025). The efficacy of artificial intelligence in diabetic retinopathy screening: a systematic review and meta-analysis. Int J Retina Vitreous.

[11] Kummerle D, Beals D, Simon L, Rogers F, Pogroszewski S (2025). Revolutionizing diabetic retinopathy screening: integrating AI-based retinal imaging in primary care. J CME.

[12] Rani PK, Kalavalapalli D, Narayanan R, Kalavalapalli S, Narula R, Sahay RK, Deo S (2025). SMART (artificial intelligence enabled) DROP (diabetic retinopathy outcomes and pathways): study protocol for diabetic retinopathy management. PLoS One.

[13] Xu X, Zhang M, Huang S, Li X, Kui X, Liu J (2024). The application of artificial intelligence in diabetic retinopathy: progress and prospects. Front Cell Dev Biol.

[14] Wang Z, Li Z, Li K, Mu S, Zhou X, Di Y (2023). Performance of artificial intelligence in diabetic retinopathy screening: a systematic review and meta-analysis of prospective studies. Front Endocrinol (Lausanne).

[15] Mathenge W, Whitestone N, Nkurikiye J (2022). Impact of artificial intelligence assessment of diabetic retinopathy on referral service uptake in a low-resource setting: the RAIDERS randomized trial. Ophthalmol Sci.

[16] Moya-Sánchez EU, Sánchez-Perez A, Nanclares Da Veiga R (2025). Design and validation of a responsible artificial intelligence-based system for the referral of diabetic retinopathy patients [PREPRINT]. arXiv.

[17] Tymchenko B, Marchenko P, Spodarets D (2020). Deep learning approach to diabetic retinopathy detection [PREPRINT]. arXiv.

[18] Kong M, Song SJ (2024). Artificial intelligence applications in diabetic retinopathy: what we have now and what to expect in the future. Endocrinol Metab (Seoul).

[19] Malerbi FK, Melo GB (2022). Feasibility of screening for diabetic retinopathy using artificial intelligence, Brazil. Bull World Health Organ.

[20] Sosale B, Aravind SR, Murthy H, Narayana S, Sharma U, Gowda SG, Naveenam M (2020). Simple, mobile-based artificial intelligence algorithm in the detection of diabetic retinopathy (SMART) study. BMJ Open Diabetes Res Care.

[21] Haddaway NR, Page MJ, Pritchard CC, McGuinness LA (2022). PRISMA2020: an R package and Shiny app for producing PRISMA 2020-compliant flow diagrams, with interactivity for optimised digital transparency and open synthesis. Campbell Syst Rev.

